# Delerium Tremens, History and Treatment of, with Emetics

**Published:** 1839-03-09

**Authors:** 


*Delerium Tremens, History and Treatment of, with Emetics*, by Stoll, in 1778.—The ancient writers have confounded under the general term *phrenzy* various affections, which the moderns have discriminated, and which they have sometimes described as new and unnoticed diseases. Among these we may place that bizarre affection, which was well portrayed by the English physician Sutton under the name of *delirium tremens*, and which, he shewed, was in almost every case occasioned by the abuse of spirituous liquors.

In lately perusing the works of Stoll, I (Professor Forget of Strasburg) was much struck with the very exact and faithful delineation of this disease, in the Chapter on the Causes and Seat of Phrenzy.

The report of the following three cases will interest every medical reader, not only by the accuracy of the description, but also by the judicious therapeutical instructions recommended by the author.

*Case* 1. A middle-aged man, of a robust constitution, was admitted into the hospital at Vienna on the 13th of June, 1778. For six days he had been distressed with extreme lassitude, loss of appetite, frequently-recurring chills, and subsequently with a great trembling or shaking of the body, as if he was in the cold stage of an ague. The pulse was but little affected; the speech was quite clear, and the mind was tranquil. Dr. Stoll regarded the case as one of incipient bilious fever, and prescribed some light aperient to act upon the bowels.

During the night, however, the patient was unexpectedly seized with furious delirium; and, from this time until the hour of the morning visit, he was in a constant state of violent excitement, screaming out, and tearing every thing that he could get hold of. The pulse at this time was vibratory; and the surface of the body was covered with a copious sweat.

Three grains of emetic tartar were administered immediately; but neither vomiting or purging was induced. An hour afterwards a mixture, containing eight grains of the same salt was prescribed: one half to be taken at a dose, and the other half in half an hour, if no effect was produced. The patient, however, mistaking the medicine for wine, which he was continually desiring to have, swallowed nearly the whole of the mixture at once. He was vomited and purged briskly three times; and a copious perspiration broke out. The delirium soon subsided, and he fell asleep muttering to himself. On the following day, he awoke quite conscious, and almost entirely tranquillized in his mind.

*Case* 2. In the Autumn of 1778, we received into the hospital a man-servant, who was at the time in a state of furious excitement. Four or five days previously he had drunk a large quantity of strong beer, while heated from running; and he was soon afterwards seized with intense headache, and frequently-returning chills. He was immediately bled from the arm; but, although the blood was buffy, *no decided relief was procured.* During the course of the evening, he fell into a state of delirium: his eyes seemed to be pushed forwards, and they were constantly rolling about; he screamed out in violent paroxysms of rage; the whole surface of the body was drenched in profuse prespiration; and the pulse, though scarcely at all accelerated, was vibratory and jerking. A second venesection was practised, *but without benefit.*

On the following day, however, he was much more composed, and then Stoll saw him for the first time. He advised a small quantity of blood to be taken again from the arm: it proved to be not at all buffy now. Acidulated drinks and gentle aperients were also prescribed; and, along with these medicines, a four-ounce mixture containing six grains of tartrate of antimony. These means produced three copious stools, and also vomited him freely thrice. He became, soon afterwards, very calm, and at length fell into a profound soothing sleep, which lasted for many hours. In the course of a few days he had completely recovered.

*Remark.* The inutility of sanguineous depletions was strongly manifested in the preceding case.—(*Rev.*)

*Case* 3. A young man, who had been long addicted to excess in drinking, was seized with shivering, severe headache, frequent vomiting, and general weakness. At times, all his limbs trembled or shook, as if he was in an ague-fit. The pulse was quickened, hard, and full. He was bled and purged with cooling aperients—the blood was buffy. During the course of the night, however, he fell into a state of delirium and excessive restlessness. On the following day, there were frequent attacks of convulsions; and the delirium was almost constant. Next day he was conveyed to the hospital: he was then more tranquil, and could give some account of his illness.

While engaged however in speaking, his looks and whole demeanour indicated an excited and wavering state of the mind. He had no sooner finished his tale, than he was seized with furious delirium.

Five grains of the tartrate of antimony were given at once, and smaller doses were given at short intervals. After the emetic and purgative action of the medicine was freely induced, the state of the patient became much more composed. Although sleep did not come on for some time, his health gradually improved and was ultimately quite restored.

*General Remarks.* These three cases, reported by a most experienced physician of the last century, before the term *delirium tremens* had been heard of, will be read with interest and advantage. The efficacy of the antimonial treatment is most satisfactorily proved by the result of each case; and the recommendations of it by some recent writers are thus very beautifully confirmed.—*Med. Chir. Rev., from Bul. Gen. de Therapeutique.*

